# Intercellular transfer of small RNAs from astrocytes to lung tumor cells induces resistance to chemotherapy

**DOI:** 10.18632/oncotarget.7273

**Published:** 2016-02-09

**Authors:** Assaf Menachem, Victoria Makovski, Or Bodner, Metsada Pasmanik-Chor, Reuven Stein, Noam Shomron, Yoel Kloog

**Affiliations:** ^1^ Department of Neurobiology, Tel Aviv University, 69978 Tel Aviv, Israel; ^2^ Bioinformatics Unit, The George S. Wise Faculty of Life Sciences, Tel Aviv University, 69978 Tel Aviv, Israel; ^3^ Department of Cell and Developmental Biology, Sackler Faculty of Medicine, Tel Aviv University, 69978 Tel Aviv, Israel

**Keywords:** astrocytes, small RNA, lung tumor, intercellular transfer, chemotherapy

## Abstract

Brain metastases are resistant to chemotherapy and carry a poor prognosis. Studies have shown that tumor cells are surrounded by activated astrocytes, whose cytoprotective properties they exploit for protection from chemotherapy-induced apoptosis. The mechanism of such astrocytic protection is poorly understood. A non-mutational mechanism of resistance to chemotherapy that is receiving increased attention is the regulation of gene translation mediated by small noncoding RNAs (sRNAs), and particularly microRNAs (miRNAs). With the aim of examining the role of astrocytic sRNAs in promoting resistance of human lung tumor PC14 cells to chemotherapy-induced apoptosis, here we used a miRNA microarray to compare sRNA profiles of human lung tumor cells cultured with and without astrocytes. We found that sRNAs are transferred from astrocytes to PC14 cells in a contact-dependent manner. Transfer was rapid, reaching a plateau after only 6 hours in culture. The sRNA transfer was inhibited by the broad-spectrum gap-junction antagonist carbenoxolone, indicating that transfer occurs via gap junctions. Among the transferred sRNAs were several that are implicated in survival pathways. Enforced expression of these sRNAs in PC14 cells increased their resistance to the chemotherapeutic agent paclitaxel. These novel findings might be of clinical relevance for the treatment of patients with brain metastases.

## INTRODUCTION

According to the American Cancer Society, approximately 1.6 million new cases of cancer are reported each year in the United States, and up to 40% of those patients will develop brain metastases [1, 2]. The incidence of brain metastases has been increasing due to improved imagine techniques, such as magnetic resonance imaging, enabling early detection in asymptomatic patients, and to effective systemic treatments that can prolong life, allowing the cancer to disseminate to the brain [3]. Brain metastases are becoming a major challenge in therapy and prevention. Current therapies for brain metastases include surgery, radiotherapy and in a limited manner, chemotherapy [3]. Over recent decades it has become clear that malignancy is controlled by interaction between tumor cells and the stromal cells, connective tissue cells that support and maintain the function of an organ's parenchymal cells [4]. In cancer, stromal cells modify the neoplastic properties of the tumor cells and contribute to their proliferation [5–7]. The brain cells that correspond to stromal cells are the astrocytes, which are the most abundant of all cell types in the human brain and perform a variety of functions in the central nervous system (CNS) [8]. The communication between tumor cells and the CNS cells is crucial for tumor cells thriving and survival, and can impact their sensitivity to chemotherapy [9–11].

Brain metastases are highly resistant to chemotherapy and carry a poor prognosis [12, 13]. Until recently, the resistance of brain metastases to chemotherapeutic drugs was attributed to the presence of the blood−brain barrier (BBB) and efflux transporters such as p-glycoprotein [13, 14]. Recent data have revealed, however, that around metastatic lesions the BBB is permeable, and clinical trials combining chemotherapeutic drugs with p-glycoprotein inhibitors have failed to reverse tumor-cell resistance [13, 15, 16]. Thus, other factors in the brain microenvironment, such as astrocytes, have been suggested to contribute to this resistance. In line with this idea it was shown that tumor cells are surrounded by activated astrocytes which, by means of several mechanisms [17–19], protect tumor cells from chemotherapy-induced apoptosis in a contact-dependent manner [17–19]. They achieve this, for example, by sequestering calcium from the tumor-cell cytoplasm, or by up-regulating genes such as *BCL2L1*, *TWIST1* and *GSTA5*, which regulate cell survival [18, 19]. Alternatively, bidirectional signaling between astrocytes and cancer cells, mediated by the endothelin axis, upregulates survival genes in the tumor cells and protects them from chemotherapy [17].

Unfortunately, the clinical efficacy of chemotherapeutic drugs is limited because some tumors are resistant or develop resistance to the drugs. As a result, tumors often relapse aggressively and metastasize to distant organs, with devastating outcomes [20]. The causes of resistance to cancer drugs are thought to be linked to random genetic mutations, karyotypic changes and epigenetic changes causing alterations in gene function [21–24]. An important non-mutational mechanism of resistance to chemotherapy that has recently attracted much research attention is the regulation of gene translation mediated by small non-coding RNAs (sRNAs), in particular microRNAs (miRNAs). sRNAs are small non-coding RNA molecules that participate in gene regulation via base paring with complementary sequences within the messenger RNA, resulting in gene silencing [25, 26]. There are several classes of sRNAs including miRNAs, siRNAs and Piwi-interacting RNAs (piRNAs) [27]. Over the last few years it has become increasingly clear that abnormal miRNA expression plays an important role in anti-cancer drug resistance [28–32]. Several studies have demonstrated abnormal expression of miRNAs in brain tumors. Some miRNAs were found to be upregulated and others were downregulated [33]. For example, miR-17 is upregulated in glioma tissues and is associated with advanced pathological stage and with poor survival [34]. Another study has shown that reducing miR-17 increases cell viability and apoptotic activity [35].

It is well established by now that sRNAs act non-autonomously, with signals spreading from cell to cell [36]. They were shown, for example, to be spread by exosomes [37–39]. An additional mechanism, demonstrated for the first time by our group, is transfer mediated by cell-to-cell contact [40]. This study showed that both endogenously and virally encoded miRNAs are transferred from B to T cells [40]. The acquired miRNAs were functional, and acted by down-regulating the expression of their specific targets in the adopting T cells. Transfer of functional sRNAs between cells was also demonstrated in other cell systems, such as heart cells and human glioma cells [41, 42]. Sharing of miRNAs between cells was found to be important in interactions between cells in the tumor microenvironment and cancer cells. Thus, for example, miRNAs acquired by breast cancer cells from bone-marrow stromal cells can elicit cell-cycle quiescence in the recipient cells through targeting of *CXCL12* [43]. Several mechanisms have been proposed to mediate miRNA transfer. These include the exosomes, [44] and gap junctions [41–43]. In the brain, oncosomes, loaded with proteins, DNA and miRNAs, are transferred from one cell to other, and can affect the recipient cell's physiology, tumor proliferation, angiogenesis and invasion [33].

Our understanding of the interaction between tumor cells and the microenvironment has improved greatly over the last few years, but we still have only limited knowledge of how tumor cells and cells in their surrounding microenvironment affect each other by sRNA exchange or how such exchange contributes to malignancy. In the present study we focused on the potential transfer of sRNAs from astrocytes to metastatic lung tumor cells and its outcome for resistance of the tumor cells to chemotherapy. Our experimental system was based on the co-culturing of conditioned immortalized mouse astrocytes (H-2K b-tsA58 mice [45]; hereafter ‘astrocytes’) with the human lung adenocarcinoma PC14 cell line. Studies based on this cell system [17–19] have shown that co-culturing of astrocytes with PC14 cells provides contact-dependent protection of the tumor cells from toxicity of the chemotherapeutic drug paclitaxel (Taxol), rendering this system suitable for assessing the role of sRNA transfer in the astrocytic effect on tumor cells. It is possible that the co-cultured tumor cells might respond with less intensity to the astrocytes than the corresponding primary tumor cells. Our results showed that sRNAs are transferred from astrocytes to PC14 cells through gap junctions, and suggest that such transfer can protect tumor cells from chemotherapy. These novel findings are potentially of clinical relevance, and might lead to the development of new approaches for treating patients with brain metastases.

## RESULTS

### Astrocytes protect PC14 tumor cells from toxicity of paclitaxel

**Figure 1:**
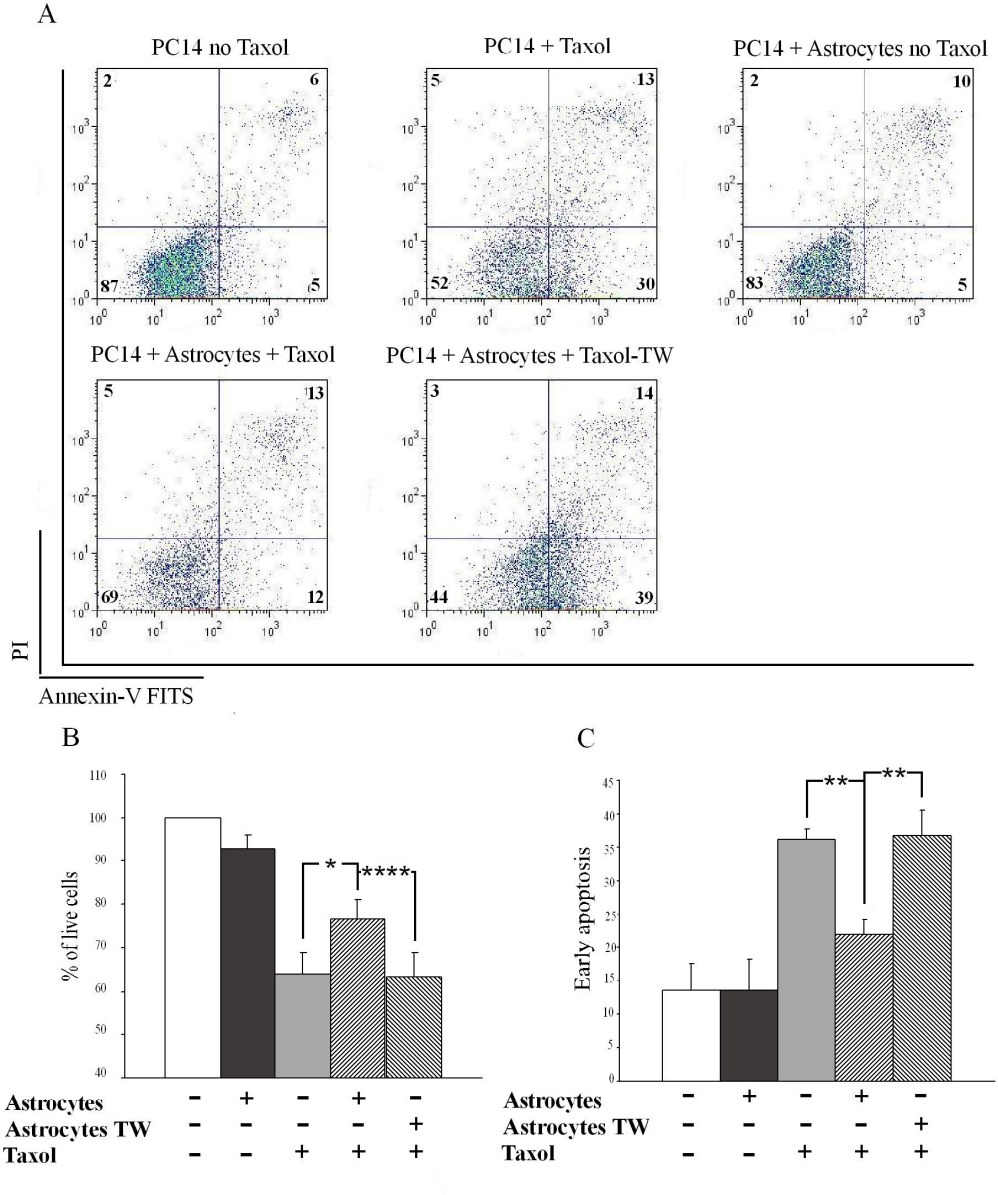
Astrocytes protect PC14 cells from Taxol-induced apoptosis in a contact-dependent manner The percentages of live and apoptotic cells were determined by FACS analysis (see Materials and Methods section). (**A**) Representative dot plot of FACS analysis of treated PC14 cells stained with annexin-V FITS and PI. PC14 singlet-cell events are distinguished from target cells by the CD340 specific marker. Numbers in the quadrants are the percentages of PC14 cells within each quadrant. (**B**, **C**) Quantitative results of FACS analysis. B. Percentage of live (low PI, low annexin V) PC14 cells cultured alone, with astrocytes, alone plus Taxol, with astrocytes in a contact-dependent manner (mixed co-culture) plus Taxol, and with astrocytes in a contact-independent manner (separated by a transwell membrane (TW)) plus Taxol. Results are expressed as percentages of untreated PC14 cells. C. Apoptotic (high annexin V, low PI) PC14 cells cultured alone, with astrocytes, alone plus Taxol, with astrocytes in a contact-dependent manner plus Taxol, and with astrocytes in a transwell (TW) plus Taxol. The results are expressed as percentage of total cells and are presented as means ± SEM. Analysis (One-way ANOVA) revealed significant differences in live cell percentages and apoptotic cells between annexin V-stained and PI-stained cells, *P* < 0.05. Post-hoc analysis was performed by Fisher's LSD: *P < 0.05, **P = 0.01, ****P < 0.0001; *n* = 4.

To examine the mechanism whereby astrocytes can promote protection of PC14 cells from apoptosis induced by the chemotherapeutic agent paclitaxel (Taxol), we incubated a co-culture of astrocytes and PC14 cells with 5 nM Taxol for 48 h and then analyzed the cells. Apoptosis was assessed by staining with annexin-V and propidium iodide (PI), a well-known method for apoptosis detection. A representative dot plot of fluorescence-activated cell-sorting (FACS) analysis of the treated cells by annexin-V-FITS and PI staining is shown in Figure 1A. PC14 cells (CD340 positive) that were cultured with astrocytes contained a significantly higher percentage of live cells than PC14 cells cultured in the absence of astrocytes or when the two cell populations were separated using a transwell (Figure 1B; mean ± SEM, 69 ± 0.8%, 52 ± 2.6%, and 36.8 ± 3.7% respectively). In addition, PC14 cells co-cultured with astrocytes demonstrated a significant decrease in the percentage of apoptotic cells (Figure 1C; mean ± SEM, 13.2 ± 0.8%, 24.4 ± 2.6%, and 36.8 ± 3.7% respectively). Taken together, these results show that astrocytes protect PC14 cells from Taxol-induced apoptosis, and that direct contact is required for this effect.

### Transfer of synthetic sRNA from astrocytes to PC14 cells

**Figure 2:**
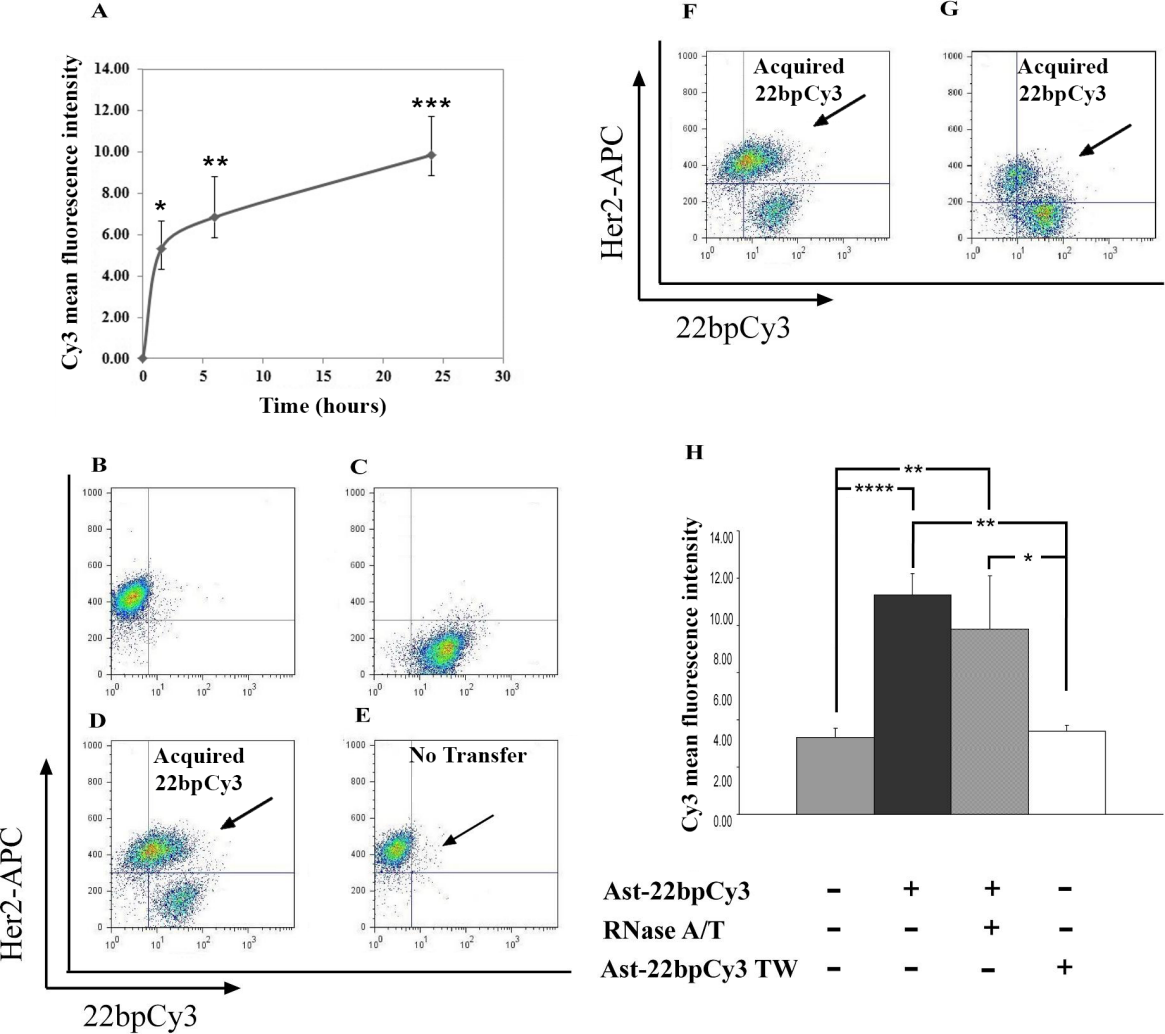
Cy3-tagged 22-bp oligonucleotides are transferred from astrocytes to PC14 cells PC14 cells (CD340 positive) containing 22bpCy3 were assayed by FACS analysis (see Materials and Methods section). (**A**) Time course of 22bpCy3 transfer from astrocytes to PC14 cells. B−G. Representative dot plots of the results of FACS analysis after the different treatments, depicting total live-cell gate records. (**B**) PC14 cells (CD340 positive) alone. (**C**) 22bpCy3-transfected astrocytes (Cy3 positive) alone; positive for Cy3. D, E. PC14 cells co-cultured with astrocytes-22bpCy3 in a contact-dependent (**D**) or contact-independent (transwell, TW) manner (**E**) F, G. Astrocytes-22bpCy3 were co-cultured with PC14 cells that were untreated (control) (**F**) or treated with RNase A/T (**G**) (**H**) Quantitative results of FACS analysis, expressed as mean fluorescence intensity (MFI) of 22bpCy3 recorded in the PC14 cells and presented as means ± SEM. Analysis (One-way ANOVA) revealed significant differences in MFI of 22bpCy3 between the groups: *P* < 0.05. Post-hoc analysis by Fisher's LSD yielded *P < 0.05, **P < 0.01, ****P < 0.0001; *n* = 5.

To examine whether sRNAs can be transferred from astrocytes to PC14 cells, we co-cultured astrocytes-22bpCy3 (astrocytes transfected with synthetic sRNA analogous to mature sRNA conjugated with Cy3 fluorophore) with PC14 cells. After 1.5 h, 6 h, or 24 h, 22bpCy3-containing PC14 cells (CD340 positive) were identified by FACS analysis. As shown in Figure 2A, after 1.5 h there was already a significant increase in the mean fluorescence intensity (MFI) of Cy3 fluorophore in the PC14 cells cultured with astrocyte-22bpCy3 compared to PC14 cells cultured alone, and this difference gradually increased after 6 h and 24 h (mean ± SEM, 5.3 ± 1.3, 6.9 ± 2.0, 9.8 ± 1.9 at 1.5 h, 6 h, and 24 h respectively).

**Figure 3:**
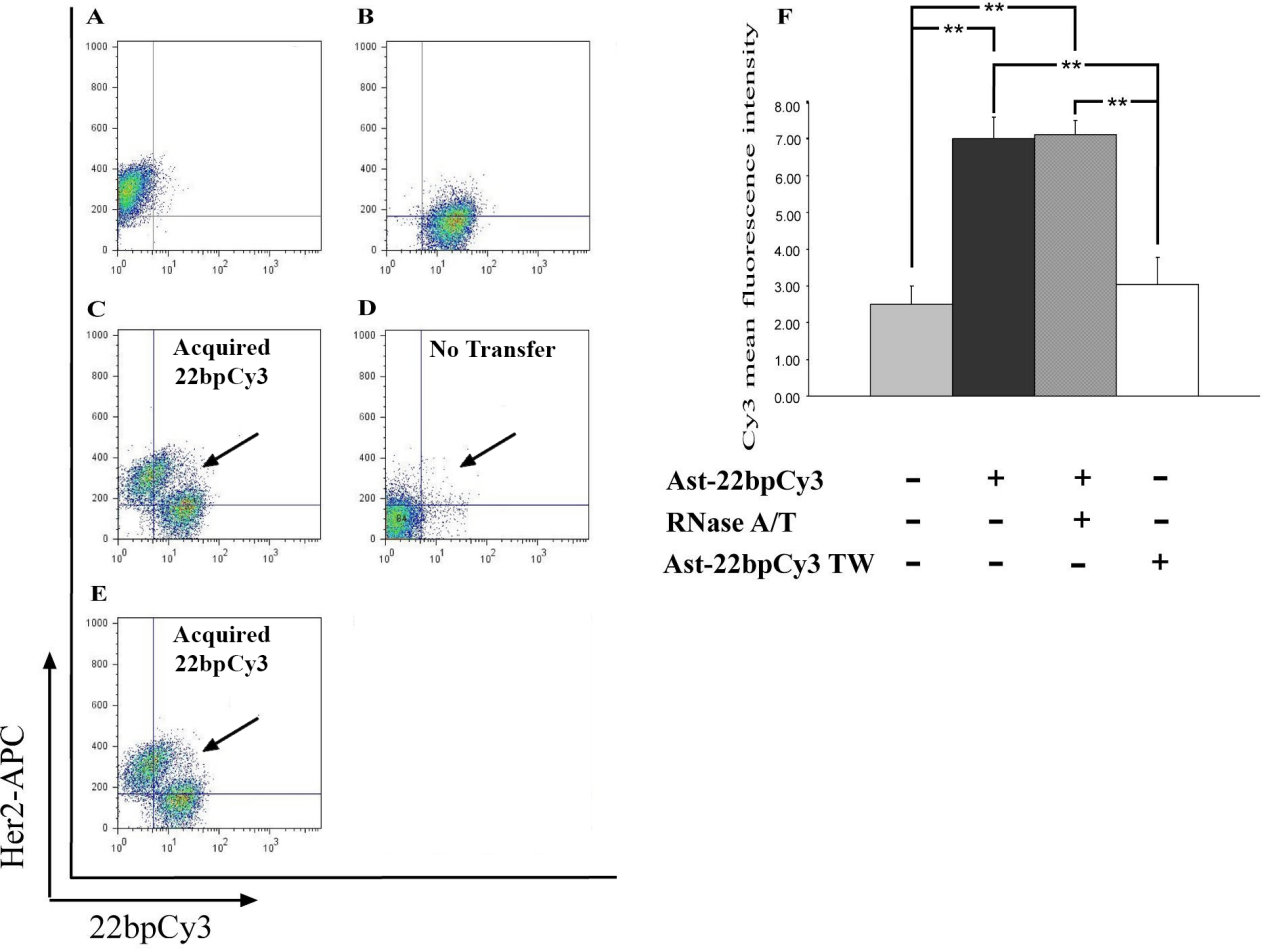
Cy3-tagged 22-bp oligonucleotides are transferred from astrocytes to MDA-MB-231 cells Astrocytes-22bpCy3 were co-cultured with MDA-MB-231 cells for 6 h. MDA-MB-231 cells (CD340 positive) containing 22bpCy3 were assayed by FACS analysis (see Materials and Methods section). (**A**−**E**) Representative dot plots of the results of FACS analysis after the different treatments, depicting total live-cell gate records A. MDA-MB-231 cells (positive for CD340) alone. B. 22bpCy3-transfected astrocytes (Cy3-positive) alone. C, D. MDA-MB-231 cells co-cultured with astrocytes-22bpCy3 in a contact-dependent (C) or contact-independent (transwell, TW) manner (D). E. MDA-MB-231 cells co-cultured with astrocytes-22bpCy3 treated with RNase A/T. (**F**) Quantitative results of the FACS analysis, expressed as MFI of 22bpCy3 recorded in the MDA-MB-231 cells and presented as means ± SEM. Analysis (One-way ANOVA) revealed significant differences in MFI of 22bpCy3 between the groups: *P* < 0.01. Post-hoc analysis by Fisher's LSD yielded **P < 0.01; *n* = 5.

To further assess the specificity of the 22bpCy3 transfer and its contact dependency, we measured the MFI of Cy3 fluorophore in PC14 cells co-cultured with astrocytes-22bpCy3 for 6 h using a transwell (contact-independent) or in a mixed co-culture (contact-dependent) manner. A representative dot plot of FACS analysis depicting total live-cell gate records is shown in Figure 2B−2E and quantification of those results in Figure 2H. After 6 h of co-culturing, the MFI of Cy3 fluorophore was significantly higher in PC14 cells that were grown in a mixed culture (i.e., in direct contact) with astrocytes-22bpCy3 than in PC14 cells cultured alone or separated by a transwell membrane from the astrocytes-22bpCy3 (Figure 2H; mean ± SEM, 9.3 ± 0.9, 3.3 ± 0.4, and 3.5 ± 0.3 respectively), indicating that PC14 cells did not acquire the synthetic sRNA when the two cell populations were not in direct contact (Figure 2F−2G). Thus, sRNA transfer was mediated not by secreted particles (such as exosomes), but rather through direct contact between the two cell populations. To confirm that the synthetic sRNA was transferred from astrocyte cytoplasm, we treated the astrocytes-22bpCy3 with RNase A/T to degrade any synthetic sRNA residues that might be attached to astrocyte membranes. Previous studies showed that RNase A/T degrade sRNA [39] although in some sRNA species under some condition e.g., treatment of pH10, membrane detergent this effect may be partial [46]. As shown in Figure 2F–2G, PC14 cells co-cultured with RNase A/T-treated astrocytes-22bpCy3 acquired a large fraction of the synthetic sRNA, which did not differ significantly from the MFI values of Cy3 fluorophore in the PC14 cells / astrocyte-22bpCy3 co-cultures that were not treated with RNase A/T (Figure 2H; mean ± SEM, 9.3 ± 0.9 and 7.8 ± 2.3, respectively To further assess the generality of sRNA transfer from astrocytes to cancer cells, we used the same procedure to examine the transfer of 22bpCy3 from astrocytes to MDA-MB-231 cancer cells. We found that 22bpCy3 was also transferred from astrocytes-22bpCy3 to MDA-MB-231 cells, and that such transfer was contact dependent (Figure 3).

### Transfer of endogenous sRNAs from astrocytes to PC14 cells

**Figure 4:**
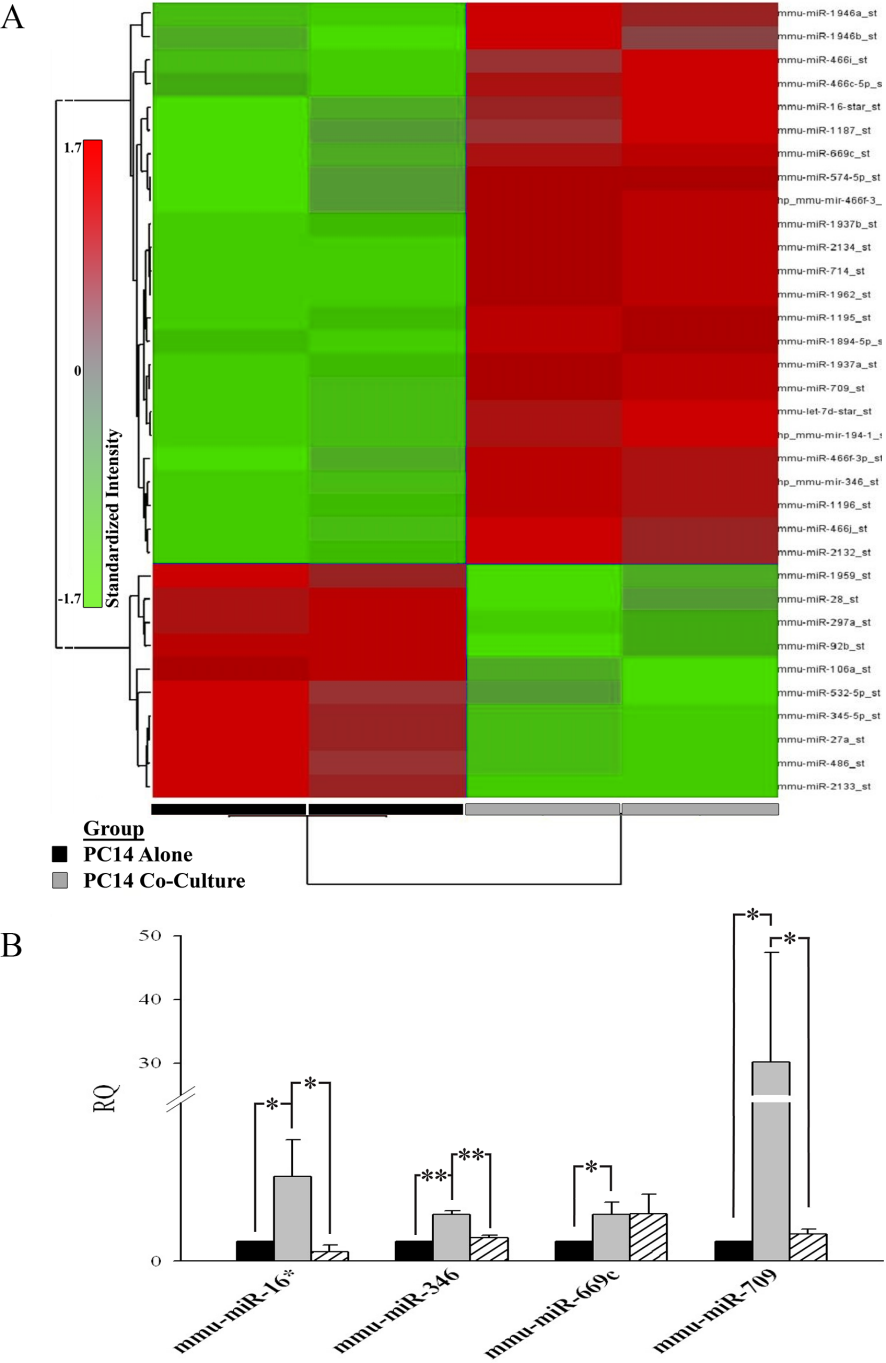
Small RNAs are transferred from astrocytes to PC14 cells Levels of mouse-specific sRNA in PC14 cells cultured alone were compared with the levels in PC14 cells co-cultured with astrocytes in the mixed co-cultures. (**A**) Heat map of miRNA microarray (two independent experiments for each treatment). Mouse-specific sRNAs (*n* = 20) whose expression was higher in PC14 cells co-cultured with astrocytes than in PC14 cells cultured alone are depicted. (**B**) Microarray results were confirmed by qPCR of the indicated miRNAs (see Materials and Methods section). Black bar, PC14 cultured alone; grey bar, PC14 cells co-cultured with astrocytes in a contact-dependent manner; striped bar, PC14 cells co-cultured with astrocytes in a contact-independent manner (transwell, TW). Results are presented as means ± SEM. Analysis (One-way ANOVA) revealed significant differences in sRNA expression between the groups: *P* < 0.05. Post-hoc analysis by Fisher's LSD yielded *P < 0.05, **P < 0.01; *n* = 3; mmu, *Mus musculus*.

Having demonstrated the transfer of synthetic sRNAs from astrocytes to human PC14 cells, we next wanted to know whether a similar transfer would occur with endogenous sRNAs. PC14 cells were co-cultured with mouse astrocytes in a contact-dependent manner for 6 h, and the PC14 cells (CD340 positive) were then isolated by FACS and total RNA was prepared. Concomitantly, PC14 cells were cultured in the absence of astrocytes or were co-cultured with mouse astrocytes in a contact-independent manner (transwell), and RNA was prepared. To identify sRNAs transferred from mouse astrocytes to human PC14 cells, we determined the total sRNA profile by miRNA microarray of the cells used in these three treatments, and identified the transferred mouse astrocytic sRNAs by searching for non-conserved mouse sRNAs in co-cultured human PC14 cells but not in human PC14 cells cultured alone. The miRNA microarray revealed significant differences in 67 sRNA levels in PC14 cells co-cultured with astrocytes for 6 h in a contact-dependent manner compared to those found in PC14 cells cultured in the absence of astrocytes (fold-change (FC) difference ≥ 1.5). Of those 67 sRNAs, 20 were mouse sRNAs that were up-regulated in the co-cultured PC14 cells (Figure 4A, FC difference ≥ 1.7), indicating that sRNAs were indeed transferred from mouse astrocytes to the human PC14 cells. The transferred sRNAs are listed in Table 1. We confirmed our results by performing quantitative RT-PCR (qPCR, Figure 4B) on selected sRNAs. Notably, miRNA microarray revealed no significant differences in miRNA levels between PC14 cells co-cultured with astrocyte in a contact-independent manner (transwell) and PC14 cells cultured alone (data not shown). We also used qPCR to support our findings that the selected sRNAs were indeed expressed in the mouse astrocytes (data not shown).

**Table 1:** List of non-conserved mouse sRNAs found in co-cultured PC14 cells

Small RNA	*P* value	Fold change
mmu-miR-2134	7.42E-05	44.7597
mmu-miR-1196	0.00671439	16.8571
mmu-miR-1195	0.00105692	15.1575
mmu-miR-709	0.00259631	12.3438
mmu-miR-1894-5p	0.0001579	6.08979
mmu-miR-1946a	0.00733713	5.59805
mmu-miR-714	0.000206816	3.95919
mmu-miR-1962	7.80E-06	3.33373
mmu-miR-1937b	0.000172545	2.95365
mmu-miR-1937a	0.000784034	2.83139
mmu-miR-1187	0.0325853	2.74294
mmu-miR-16-star	0.0190219	2.46428
mmu-miR-2132	0.029467	2.42419
mmu-miR-669c	0.0155178	2.21774
mmu-miR-466j	0.026805	2.16929
mmu-miR-466c-5p	0.0241965	1.98764
hp_mmu-mir-346	0.0142571	1.83521
mmu-let-7d-star	0.00304475	1.80533
mmu-miR-466f-3p	0.0337132	1.7063
mmu-miR-1946b	0.0323624	1.70281

The miRNA microarray reveals a significant increase in levels of 20 sRNAs in PC14 cells co-cultured for 6 h with astrocytes in a contact-dependent manner (mixed cultures) compared to PC14 cells cultured for 6 h in the absence of astrocytes (*P* < 0.05 by *t*-test: fold-change (FC) difference ≥ 1.7).

### Transfer of sRNAs from astrocytes to PC14 cells is mediated by gap junctions

**Figure 5:**
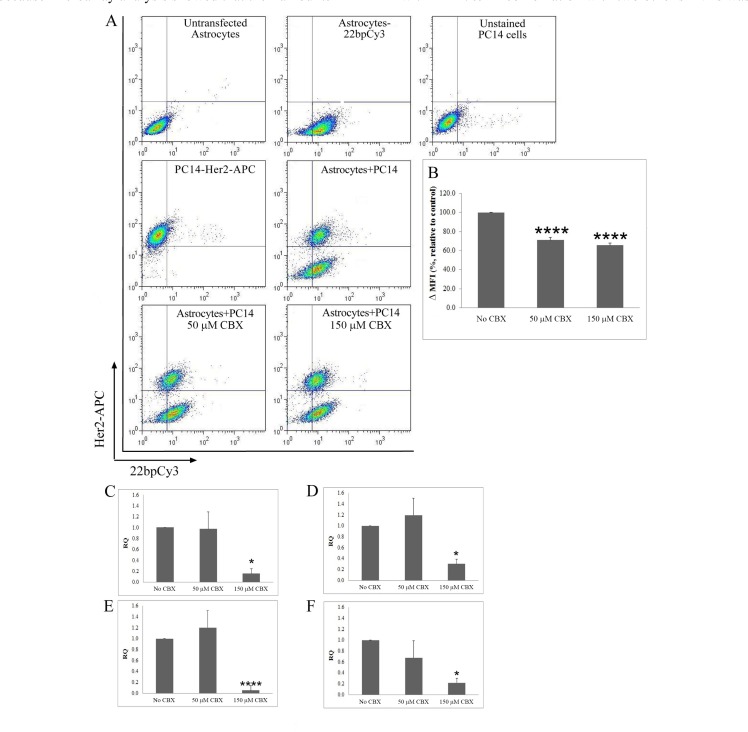
Inhibition of gap-junction communication and analysis of intercellular transfer of endogenous sRNAs (**A**, **B**) The gap-junction inhibitor CBX inhibits transfer of 22bpCy3 from astrocytes to PC14 cells. PC14 cells (CD340 positive) containing 22bpCy3 were assayed by FACS analysis (see Materials and Methods section). A. Representative dot plots of the results of FACS analysis after the different treatments, depicting total live-cell gate records. B. Quantitative results of FACS analysis expressed as MFI of 22bpCy3 recorded in PC14 cells cultured with astrocytes-22bpCy3 that were treated with CBX (50 μM/150 μM) or untreated (control). CBX inhibited the transfer of endogenous sRNA from the astrocytes to the PC14 cells. C−F. Levels of mmu-miR-16* (**C**), mmu-miR-709 (**D**), mmu-miR-1195 (**E**) and endo-siRNA-1196 (**F**) were measured in PC14-GFP cells by qPCR (see Materials and Methods section). Results are presented as means ± SEM. Analysis (One-way ANOVA revealed significant differences in MFI of 22bpCy3 between the groups: *P* < 0.05. Post-hoc analysis by Fisher's LSD yielded *P < 0.05, ****P < 0.0001; *n* = 4.

Having demonstrated the transfer of sRNAs from astrocytes to cancer cells and shown that such transfer requires direct contact between the two cell types, we next examined whether this transfer is mediated via gap-junction communication. PC14 cells and astrocytes-22bpCy3 were each pretreated for 16 h with different concentrations (50−150 μM) of Carbenoxolone (CBX), a well-known gap-junction inhibitor. The cells were then co-cultured for 6 h in the continuing presence of CBX. After treatment with 50 μM or 150 μM CBX, FACS analysis (Figure 5A) revealed a significant decrease in the MFI of Cy3 fluorophore in the PC14 cells (29% and 35%, respectively) compared to untreated cells (Figure 5B). These results indicated that CBX inhibits transfer of the Cy3-labeled synthetic sRNA from astrocytes-22bpCy3 to PC14 cells.

Next, we examined the effect of CBX on the transfer of endogenous sRNAs from astrocytes to PC14 cells. The sRNAs chosen for this analysis were mmu-miR-16*, mmu-miR-709, mmu-miR-1195 and endo-siRNA-1196, all shown on microarray analysis to exhibit marked differences in fold change, and whose reported gene targets may have relevant protective activity, for example in connection with apoptosis, cell cycle, and p53-signaling pathways (Qiagen's Ingenuity^®^ Pathway Analysis). Astrocytes and PC14-GFP cells were pretreated with 50 μM or 150 μM CBX for 16 h, and were then co-cultured for 6 h in the continuing presence of CBX, after which PC14 cells (GFP-positive) were isolated by FACS and their total RNA was prepared. Levels of mmu-miR-16*, mmu-miR-709, mmu-miR-1195 and endo-siRNA-1196 were assayed by qPCR. sRNA levels were not affected by treatment with 50 μM CBX However, treatment with 150 μM CBX significantly decreased mmu-miR-16* levels by 80%, mmu-miR-709 levels by 70%, mmu-miR-1195 levels by 82%, and endo-siRNA-1196 levels by 78% compared to CBX-untreated cells (Figure 5C−5F). These findings suggested that the transfer of sRNAs from astrocytes to PC14 cells is mediated by gap-junction communication.

### Combined enforced expression of miR-709 with two other sRNAs protects PC14 cells from Taxol toxicity

**Figure 6:**
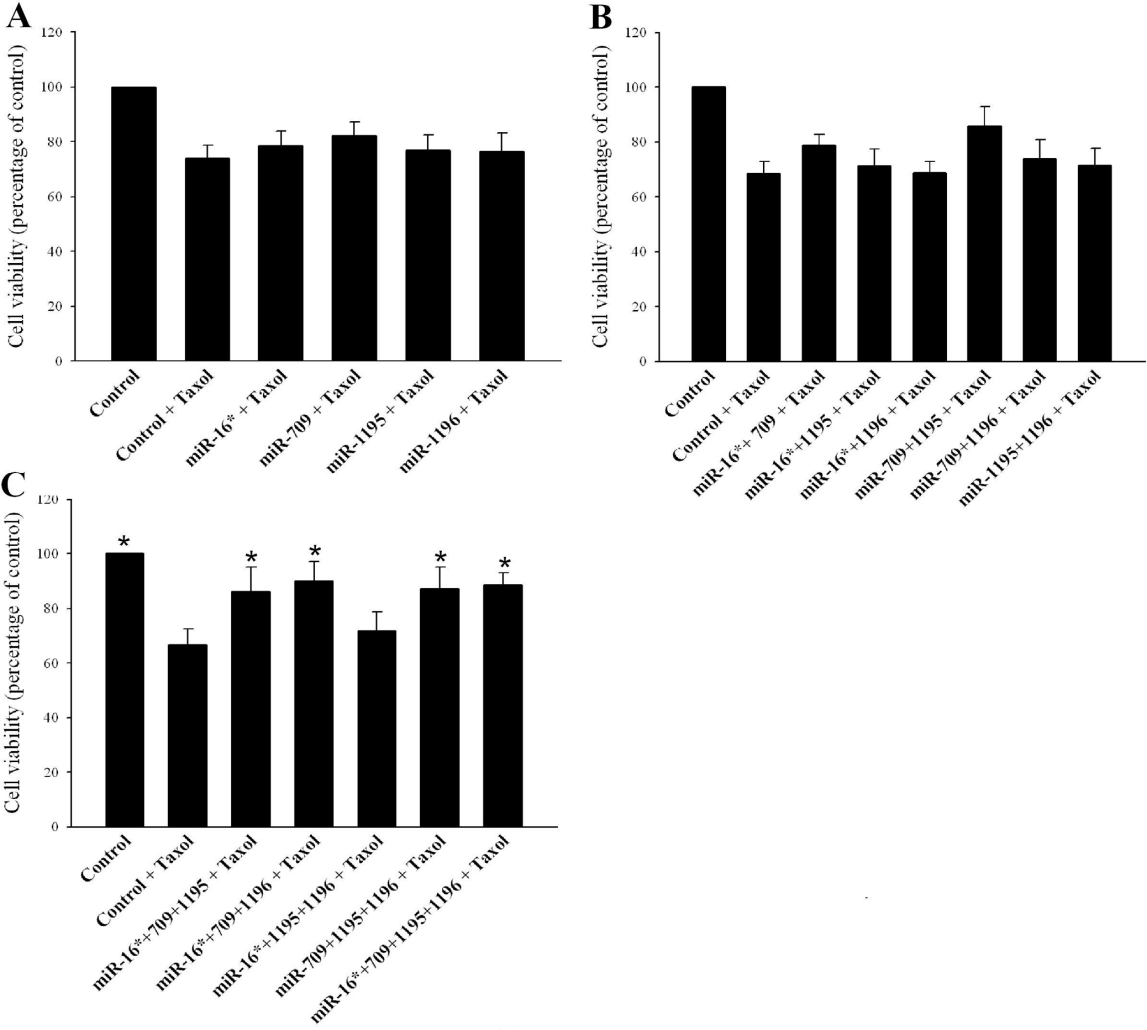
Combined enforced expression of miR-709 with two other sRNAs protects PC14 cells from Taxol-induced toxicity (**A**, **B**) Transfection with one or two sRNAs did not increase cell viability compared with control cells transfected with either mimic negative control or empty miR-Vec plasmid or both. (**C**) Transfection with miRNA-709 in combination with two sRNAs or all four sRNAs significantly increased cell viability compared to controls. Results are presented as means ± SEM. Analysis (One-way ANOVA) revealed significant differences between the groups: *P* < 0.05. Post-hoc analysis by Fisher's LSD yielded *P < 0.05; *vv* = 5.

To further support the notion that sRNAs transferred from astrocytes promote a protective effect of astrocytes against chemotherapy, we next examined whether the transferred sRNAs identified here showed the potential for promoting a protective effect against Taxol in PC14 cells. To this end we exogenously expressed mmu-miR-16*, mmu-miR-709, mmu-miR-1195 and endo-siRNA-1196 sRNAs in PC14 cells. The mmu-miR-16*, mmu-miR-1195, and endo-siRNA-1196 were expressed in the PC14 cells via the miR-Vec miRNA-expressing system [47] and mmu-miR-709 was expressed via miRNA mimic. PC14 cells were transiently transfected with each of these sRNAs or with their combinations, and 24 h later the transfected cells were treated with Taxol (25 nM) for 48 h and their viability was examined by the MTT assay. As a control, PC14 cells were transfected with a mimic negative control, empty miR-Vec plasmid, or both. Transfection with one sRNA or with two sRNAs did not increase cell viability compared to control (Figure 6A−6B). However, transfection with miR-709 plus two sRNAs, or transfection with all four sRNAs (miR-16* + miR-709+ miR-1195, miR-16* + miR-709 + endo-siRNA-1196, miR-709 + miR-1195 + endo-siRNA-1196, and miR-16* + miR-709 + miR-1195+ endo-siRNA-1196), significantly increased cell viability compared to controls (mean ± SEM, 86% ± 11%, 90% ± 6.9%, 87% ± 8%, 88.6% ± 4.5%, and 66.7% ± 5.8% respectively; Figure 6C). Transfection with miR-16*+ miR-1195+ endo-siRNA-1196 did not protect PC14 cells relative to control (76.7% ± 6.5% and 66.7% ± 5.8% respectively; Figure 6C). Taken together, these results suggest that enforced expression of miR-709 combined with two other sRNAs can promote a protective effect against Taxol toxicity in PC14 cells.

## DISCUSSION

The aim of this study was to examine the possibility that the transfer of sRNAs from astrocytes to metastatic tumor cells underlies the mechanism whereby astrocytes protect brain metastatic tumors from the toxicity of chemotherapy. We focused on the transfer of sRNAs from astrocytes to PC14 cells, a cell line of aggressive human lung adenocarcinoma, and to MDA-MB-231, an aggressive human breast adenocarcinoma cell line. Both cell lines are known to metastasize to the brain [19, 48]. Our results showed that sRNAs are indeed transferred from astrocytes to these cancer cells, that the transfer is contact-dependent and most probably occurs through gap junctions, and that the combined expression of selected transferred sRNAs in PC14 cells was found to increases their resistance to chemotherapy. Collectively, these results suggested that astrocytes protect brain metastatic cancer cells from chemotherapy by transferring sRNAs to the cancer cells, where they promote protective activity. Previous studies have also shown that astrocytes promote cancer cell protection [18, 19]. However, whether the transferred sRNAs regulate the processes by which astrocytes protect tumor cells from chemotherapy, or act independently of them, is not yet known. To examine the possibility that the transferred sRNAs protect the cancer cells from chemotherapy, we focused on four sRNAs (mmu-miR-16*, mmu-miR-709, mmu-miR-1195, and endo-siRNA-1196), all found here to be transferred from astrocytes to PC14 cells. These sRNAs were chosen because microarray analysis showed that their amounts in the tumor cells were high relative to the other transferred sRNAs, and also because their reported gene targets may be of relevance for a protective role, for example in apoptosis, in cell cycle, or in p53 signaling. We found that enforced expression with one or two sRNAs did not suffice to increase cell viability, but that enforced expression with miR-709 in combination with two other sRNAs was sufficient to induce the protective effect, suggesting that this miRNA might play an important role in the protective effect mediated by the astrocytes. These results are in line with previous results suggesting that miR-709 can act as an oncomiR [49–51]. Thus, in hepatocellular carcinoma miR-709 is up-regulated and promotes proliferation, migration, and invasion by targeting glypican-5 (GPC5), a tumor suppressor protein that is highly expressed in lung adenocarcinoma tissue relative to its expression in normal lung tissue [49, 52]. In addition, miR-709 has been shown to regulate the biogenesis of miR-15a and miR-16-1 through suppression of pre-miR-15a/16-1 maturation, suggesting a role for miR-709 in the regulation of apoptosis through the miR-16/*Bcl 2* pathway [51]. Moreover, DNA damage in germ cells up-regulates miR-709 expression, which in turn targets the mRNA of *BORIS* (brother of the regulator of imprinted sites) to counteract aberrant DNA hypomethylation [50]. Another study showed that miR-709 acts as a suppressor of oncogenesis. Reduced levels of miR-709 are required for the initiation and maintenance of human T cell acute lymphoblastic leukemia [53]. Our results showed that mmu-miR-1195, endo-siRNA-1196 and mmu-miR-16* may also contribute to the protective effect, although only when administered in combination with miR-709. miR-1195 is regulated by the NK2 homeobox 1 (NKX2-1) [54]. NKX2-1 has both oncogenic and tumor-suppressor functions depending on the cell context [55]. In lung cancers, NKX2-1 is amplified and may participate in the pulmonary tumorigenic process [56]. sRNA-1196 is a DICER-dependent and DGCR8-independent sRNA that was recently reclassified as an endo-siRNA [57]. Endo-siRNAs are a class of small RNAs generated by the sequential cleavage of the endonuclease DICER, independently of DROSHA, of long double-stranded RNA molecules. A role for endo-siRNAs has been documented in several biological systems, such as development and stress response [58]. Their role in cancer, however, remains largely unknown. With respect to miR-16*, precursor miRNAs are processed in the cytoplasm by the endonuclease DICER, producing a mature miRNA and a passenger strand. The mature miRNA that regulates gene expression is the guide strand (miR-#-5p). The passenger strand (miR-#-3p/miRNA*) is believed to be degraded and inactivated [59]. Accumulating evidence suggests, however, that the miRNA* strand can regulate gene expression [60]. In prostate cancer cells, for example, miR-17-3p functions as an oncogene [60]. The human genome does not contain obvious homologues of miR-16*, miR-1195 and Endo-siRNA-1196; it does, however, contain target sequences of these sRNAs in some of their 3′UTR mRNAs. miR-709 is an exception in that its seed is identical to that of the human miR-1827. miR-1195 is also partially homologous with the human miRNA hsa-miR-584-3p.

We found that the mechanism whereby sRNAs are transferred from astrocytes to PC14 cells involves direct interaction between the cells, and that the sRNAs are probably transferred via gap junctions between the two cell types. The need for direct cell-cell contact was shown by the fact that transfer of 22bpCy3 from astrocytes to PC14 cells was not detectable when donor and acceptor cells were separated by a transwell membrane. Further support came from the miRNA microarray analysis, since we could not detect the presence of mouse-derived sRNAs in PC14 cells co-cultured with astrocytes in the transwell experiment. We examined the possibility of transfer via gap junctions by assessing the effect of CBX, a well-known gap-junction antagonist on the sRNA transfer. CBX is a glycyrrhetinic acid derivative with a steroid-like structure that has diverse pharmacological activities. It influences endogenous glucocorticoids and has been used to treat peptic, esophageal and oral ulceration, and inflammation. The principal side effects of CBX are sodium retention with suppression of the renin-angiotensin-aldosterone system [61]. CBX is also known as a modestly potent, water-soluble blocker of gap junctions [62, 63]. We cannot exclude the possibility that CBX act on the sRNAs transfer by other mechanism(s) besides affecting gap junction. However, since numerous studies have shown that CBX affects gap junctions, [17−19, 64−67] we favor more the possibility that its effect is mediated via the gap-junction. CBX significantly reduced the transfer of 22bpCy3 as well as of mmu-miR-709, mmu-miR-1195, endo-siRNA-1196, and mmu-miR-16*. Notably, CBX was more effective in inhibiting the transfer of the endogenous sRNAs than 22bpCy3. One possible explanation for this difference is that the concentration of 22bpCy3 in the transfected astrocytes is much higher than endogenous sRNAs and therefore higher concentration of CBX is required to inhibit its transfer compared to the concentration needed to inhibit the transfer of endogenous sRNAs. Another potential explanation is that 22bpCy3 might be transferred from astrocytes to tumor cells via additional mechanisms besides gap junctions. Nonetheless, these results strongly suggested that sRNAs are transferred from astrocytes to cancer cells via gap junctions. This assumption is further supported by our finding that both astrocytes and PC14 cells expressed gap junction proteins (data not shown). These findings are in line with a previous study showing that inhibition by CBX of the protective effect of astrocytes co-cultured with Taxol-treated cancer cells is mediated by gap junctions [18].

Altogether, our results suggest that cell-to-cell transfer of sRNAs from astrocytes to tumor cells serves as a mechanism for promoting the resistance of brain metastatic tumors to chemotherapy. These findings may therefore lead to the development of promising new therapeutic approaches that target such transfer for the treatment of patients with brain metastases.

## MATERIALS AND METHODS

### Cell cultures and reagents

Human lung adenocarcinoma cell line PC14 [19, 48] and human breast adenocarcinoma cell line MDA-MB-231 were maintained as monolayer cultures as described in detail elsewhere [19] at 37°C in a humidified 5% CO_2_ incubator. Conditioned immortalized mouse astrocytes were isolated from neonatal mice homozygous for a temperature-sensitive SV40 large tumor (T) antigen (H-2Kb-tsA58 mice; CBA/Ca × C57Bl/10 hybrid; Charles River Laboratories) and established in culture as described in detail elsewhere [45]. Cells were maintained at 33°C in a humidified 8% CO_2_ incubator.

### Chemoprotection experiments

PC14 cells were cultured with astrocytes at a 1:1 ratio and treated with Taxol (5 nM) for 48 h. The cells were then trypsinized, washed once with PBS and resuspended in PBS (100 ul). PC14 was immunofluorescently stained with APC anti-human CD340 (erbB2/HER2) Antibody (Ab; Biolegend), for 15 min at room temperature (RT). To assess chemoprotection, the percentages of live cells and apoptotic cells were quantified.

### Apoptosis assay

To measure apoptosis we used the MEBCYTO^®^ Apoptosis Kit (annexin V-fluorescein isothiocyanate (FITC) and propidium iodide (PI); MBL International) according to the manufacturer's instructions. Percentages of live and apoptotic cells were measured by flow cytometry using a single laser-emitting excitation light at 488 nm. PC14 singlet-cell events were distinguished from target cells by their APC anti-human CD340 specific marker. APC signal was detected by excitation with a 633 nm laser through a 660/20 bandpass filter. Data collected from ∼10,000 single-cell events were analyzed.

### Synthetic small RNA transfection

Astrocytes (2.5 × 10^5^, 6-well tissue culture plate) were transfected with Cy^™^3 dye-labeled Pre-miR^™^ Negative Control #1 (22bpCy3, Ambion) using TransIT-siQUEST Transfection Reagent (Mirus), according to the manufacturer's instructions. A complex of TransIT-siQUEST (6 μl) and Cy3^™^-labeled-miR (1 μl, 50 μM) was diluted in 250 μl Opti-MEM I Reduced-Serum Medium and dripped gently onto the astrocytes.

### Co-cultures and analysis of intercellular transfer of synthetic sRNA by FACS

For intercellular transfer of synthetic sRNA we used 22bpCy3 as a transfer marker. Donor astrocytes were transfected with 22bpCy3 as described above, and 24 h after transfection the cells were thoroughly washed to remove surplus 22bpCy3 left from the original transfection prior to co-culturing. Cells of the PC14 or MDA-MB-231 cell line (4 × 10^5^) were added to the 22bpCy3-transfected astrocytes (hereafter ‘astrocytes-22bpCy3’) to obtain a 1:1 effector:target ratio and were co-cultured at 37°C in a humidified 5% CO_2_ incubator for 6 h. For FACS-based analysis of 22bpCy3 transfer, the cells were trypsinized, washed once with PBS, and resuspended vigorously in 5 mM EDTA/PBS. PC14 or MDA-MB-231 cells were immunofluorescently stained with APC anti-human CD340 Ab as described above. After labeling the cells were washed with PBS and again resuspended in 5 mM PBS/EDTA. Transfer of the synthetic sRNA from astrocytes to PC14 cells was analyzed by FACS.

### FACS analysis

For multi-parametric FACS analysis, cell samples were analyzed on a FACSCalibur™ using Cellquest™ software or on a FACSAria™ instrument using FACSDiva™ software (all from BD Biosciences). Data collected from ∼10,000 single-cell events were analyzed. PC14 singlet-cell events were distinguished from target cells by their APC anti-human CD340 specific marker. The Cy3 signal was detected by excitation with a 561 laser through a 582/15 bandpass filter, and the APC signal by excitation with a 633 laser through a 660/20 bandpass filter. An increase in mean fluorescence intensity (MFI, log mode) of 22bpCy3 recorded in PC14 cells co-cultured with astrocytes relative to PC14 cells cultured alone was used to evaluate the intercellular transfer of the synthetic small RNA. Final data analysis was performed with FlowJo software (Ashland).

### Time-course assay

To examine the transfer time course of the tagged synthetic sRNA, PC14 cells (4 × 10^5^) were co-cultured with astrocytes-22bpCy3 (2.5 × 10^5^) in a 6-well plate at 37°C, for 1.5 h, 6 h, or 24 h. At the end of the co-culturing the PC14 cells were stained with APC anti-human CD340 Ab to label PC14 cells, and analyzed for 22bpCy3 acquisition by FACS as described above.

### RNase A/T1 treatment

To confirm that the synthetic sRNA was transferred from astrocyte cytoplasm, a co-culture experiment was carried out as described above. Prior to co-culturing, astrocytes-22bpCy3 were treated for 10 min at 37°C with RNase A/T1 cocktail (0.4 μg/μl, Thermo Scientific) to degrade any synthetic sRNA residues attached to astrocyte membranes, as described elsewhere [39]. Transfer of 22bpCy3 to PC14 cells was examined by FACS as described above.

### Transwell assay

PC14 cells were prevented from direct contact with astrocytes-22bpCy3 by a semipermeable 0.2-μm-pore transwell membrane (Corning Costar). In brief, astrocyte-22bpCy3 (2.5 × 10^5^ cells in 1 ml of medium) were plated in the lower chamber and PC14 cells (4 × 10^5^ cells, in 1 ml of medium) were added in 6-well plates to the upper compartment. The cells were incubated for 6 h at 37°C. Transfer of 22bpCy3 to PC14 cells was examined by FACS as described above.

### Co-culturing and analysis of intercellular transfer of endogenous sRNAs

Astrocytes and PC14 cells were plated, separately or as co-cultures, at a tumor cell to astrocyte ratio of 1:1, onto 35-mm-diameter wells of 6-well tissue-culture multiwell dishes. The cells were incubated at 37°C in a humidified 5% CO_2_ incubator for 6 h, harvested, and stained for 15 min at RT with APC anti-human CD340 Ab to label PC14 cells and with anti-mouse GLAST-PE conjugated antibody (Miltenyi Biotec) to label astrocytes. To obtain a single-cell uspension, the cells were pretreated as described above to dissociate cell conjugates. PC14-CD340-APC cells were separated from GLAST-PE-labeled astrocytes (hereafter astrocytes-GLAST-PE) by a FACSAria™ sorter at 4°C using FACSDiva^™^ software. The PE signal was detected by excitation with a 561 laser through a 582/15 bandpass filter, and the APC signal by excitation with a 633 laser through a 660/20 bandpass filter. For each experiment, cells missing specific staining were analyzed to establish the background signal and to set gates for the sorting of positive cells. RNA was purified by TRIzol^®^ reagent (Life Technologies) according to manufacturer's instructions, and mouse sRNA levels in PC14 cells cultured in the presence or absence of astrocytes were measured by microRNA microarray.

### miRNA microarray

miRNA microarray was conducted as described previously [68]. miRNAs were extracted using TRIzol^®^. RNA quantity and quality were determined using a NanoDrop^™^ 2000 spectrophotomer (Thermo Scientific). Affymetrix GeneChip^®^ miRNA 2.0 arrays were used for genome-wide miRNA-expression analysis (15,644 probe sets for 131 organisms, including 1105 mature human miRNAs, 1105 human pre-miRNAs, 722 mature mouse miRNAs and 690 mouse pre-miRNAs) according to the instruction manual (Affymetrix, Santa Clara). Two biological repeats were used for each treatment.

### Bioinformatics analysis

Microarray analysis was performed on CEL files using Partek^®^ Genomics Suite™ software (Partek GS; Partek). Quantile normalization was performed by the Robust Multi-array Average method (RMA), followed by One-way ANOVA. Mouse sRNAs that were differentially expressed (*P* < 0.05; fold-change cutoff of 1.7) were obtained.

### miRNA-real time PCR

Total RNA was extracted from PC14 cells using TRIzol^®^ reagent. RNA quantity and quality were determined using NanoDrop™. Four additional biological repeats (total of 6 repeats) were subjected to miRNA real-time (RT)-PCR using specific TaqMan^®^ Small RNA probe sets for mmu-miR-16*, mmu-miR-709, mmu-miR-1195, endogenous siRNA-1196 (endo-siRNA, custom made, sequence: AAAUCUACCUGCCUCUGCCU), mmu-miR-346, and mmu-miR-669c (Applied Biosystems). miRNA expression is represented relative to the expression of the internal control U6-snRNA. Data were analyzed by the 2^−ΔΔCt^ method using 7300 RT-PCR system software (Applied Biosystems).

### Inhibition of gap-junction communication and analysis of intercellular transfer of endogenous sRNAs

To inhibit gap-junction communication between astrocytes-22bpCy3 and PC14-GFP cells, both cell types were separately incubated with CBX (50 μM/150 μM) for 16 h. Cells were then co-cultured for 6 h in the continuing presence of CBX at 37°C in a humidified 5% CO_2_ incubator. To obtain a single-cell suspension, cells were pretreated as described above to dissociate the cell conjugates. GFP-transfected PC14 cells were separated from astrocytes by a FACSAria™ sorter at 4°C using FA CSDiva^™^ software. Viable single cells were gated using side and forward scatter, with subsequent discrimination of doublets. GFP signal was detected following excitation with a 488 laser through a 530/30 bandpass filter. For each experiment, cells missing specific staining were analyzed to establish the background signal and to set gates for the sorting of positive cells. RNA was extracted, quantified, and subjected to miRNA RT-PCR for mmu-miR-16*, mmu-miR-709, mmu-miR-1195 and endo-siRNA-1196, as described above.

### Transfection of miRNA mimic and miR-Vec vector

PC14 cells were plated in 96-well plates (0.8 × 10^4^ cells/well). After 24 h the cells were transfected with TransIT^®^ × 2 (Mirus) with mimic-mmu-709, miR-Vec-16*, miR-Vec-1195, miR-Vec-1196 or, as controls, with either mimic negative control or with empty miRVec plasmid, a modified pMSCV-Blasticidin vector described elsewhere [47]. Cells were transfected with all possible combinations of the four miRNAs. After 24 h the cells were treated with 25 nM Taxol for 48 h. Cell viability was measured by MTT assay.

### MTTassay

Cells were incubated with MTT solution at a final concentration of 0.5 mg/ml for 1 h at 37°C, and DMSO was then added. The plates were read on a micro-ELISA reader (Mannedorf) at a test wavelength of 570 nm and a reference wavelength of 630 nm. Cell viability was calculated as the ratio of absorbance in treated cultures to absorbance in untreated control cultures.

### Statistical analysis

Results are expressed as mean values ± SEM. *P* values were calculated by One-way ANOVA. Post-hoc analysis was performed by Fisher's least significant difference (LSD) test. All analyses were performed with SPSS software.
